# Report of a Case of Poisoning by Opium—Belladonna Used as an Antidote

**Published:** 1862-10

**Authors:** Ira D. Hopkins

**Affiliations:** Utica, N. Y.


ART. III.—*Report of a case of Poisoning by Opium—Belladonna used as an antidote.*

Ida Clifford, aged twenty, was admitted into the City Hospital on the 23d of July last, at 5 o'clock P. M. I was informed that she had purchased XL grs. of opium about 9 o'clock A. M. When I arrived at 6 o'clock P. M. I found her quite insensible, breathing heavily, pupils contracted to a point, skin cold and moist; pulse was feeble and scarcely perceptible. Being confident that the time for emetics and the stomach pump had passed, it became necessary to resort to some antidote that would reach the absorbed poison in the circulation; and testimony favoring belladonna, I administered ʒi of that tinct., and ordered ʒss. every two hours. In the morning I found her quite smart.

I am, yours, &c. IRA D. HOPKINS, M. D.,

Utica, N. Y.

